# Polypharmacy, Gender Disparities, and Ethnic and Racial Predispositions in Long QT Syndrome: An In-Depth Review

**DOI:** 10.7759/cureus.46009

**Published:** 2023-09-26

**Authors:** Bruno Lima, Soha Razmjouei, Muhammad Talha Bajwa, Zoha Shahzad, Oluwasegun A Shoewu, Osama Ijaz, Pooja Mange, Shandesh Khanal, Tsion Gebregiorgis

**Affiliations:** 1 Medicine, University of Grande Rio, Rio Grande, USA; 2 Anesthesiology, Case Western Reserve University School of Medicine, Cleveland, USA; 3 Internal Medicine, Services Hospital Lahore, Lahore, PAK; 4 Internal Medicine, Fatima Jinnah Medical University, Lahore, PAK; 5 Family Medicine, Hackensack University Medical Center, Hackensack, USA; 6 Internal Medicine, K.J. Somaiya Hospital and Research Center, Mumbai, IND; 7 Internal Medicine, Lumbini Medical College, Kathmandu, NPL; 8 General Practice, Addis Ababa University Medical Faculty, Addis Ababa, ETH

**Keywords:** racial predisposition, gender disparity, schwartz score, genetic mechanisms, polypharmacy

## Abstract

Long QT syndrome (LQTS) is a complex disorder of cardiac electrophysiology. It is characterized by delayed myocardial polarization leading to QT prolongation and alterations on the ST segment and T wave visible on electrocardiogram (ECG). Syncope is a common manifestation, and torsade de pointes (TdP) can lead to sudden cardiac death. Three major LQTS genes (KCI31, KCNH2, and SCN5) lead to most of the cases of LQTS. Lifestyle modifications, beta blockers, and implantable cardioverter defibrillator (ICD) placement are the main treatments for LQTS. Polypharmacy, including QT-prolonging drugs, has been shown to worsen LQTS. The impact on potassium channels and the human ether-a-go-go-related gene (hERG) is the mechanism behind the QT interval prolongation caused by these medications. There is an increased incidence of LQTS among African-American men and women as compared to Caucasians. Women with LQTS tend to have a higher mortality rate from the condition, especially during menstruation and shortly after giving birth. Genetic testing is reserved to those patientswho exhibit either a strong clinical index of suspicion or experience persistent QT prolongation despite their lack of symptoms. Knowing the genetics, racial, and gender discrepancies can help improve patient management and a better comprehension on each case. Proper understanding of how ion channels function and their interaction with medications will lead to a better comprehension and to develop effective forms to treat those patients.

## Introduction and background

Long QT syndrome (LQTS) is a potentially life-threatening arrhythmia characterized by delayed ventricular repolarization that produces QT prolongation on electrocardiogram (ECG) and an increased risk of torsades de pointes (TdP), which can lead to cardiac events, such as syncope, cardiac arrest, and sudden cardiac death (SCD) [1]. LQTS is a rare genetic disorder and a major cause of SCD in the young that can be prevented. The prevalence of LQTS is estimated to be ∼1:2,000, with a slight female predominance [2]. The availability of medications highlights the importance of early and accurate diagnosis of LQTS [3]. LQTS presents as two congenital forms, Romano-Ward syndrome (RWS, autosomal dominant) and Jervell and Lange-Nielsen syndrome (JLNS, autosomal recessive disorder). Most commonly, QT interval prolongation is produced due to reductions in either the rapidly or the slowly activating delayed repolarization cardiac potassium (K+) current, I_Kr_ or I_Ks_. Less commonly, it results from prolonged depolarization due to a small persistent inward “leak” in heart sodium (Na+) current, INa [4]. Recently, the genes associated with LQTS linked to chromosomes 3 (LQT3), 7 (LQT2), and 11 (LQTl) were identified as SCN5A, the cardiac sodium channel gene and as human ether-a-go-go-related gene (hERG) and KvLQTl potassium channel genes. These discoveries have led to the development of gene-specific therapy for these three types of LQTS [5].

Molecular basis and genetic mechanisms

The cardiac action potential is vital to proper heart function. Beginning with the activation of the sinoatrial (SA) node, the action potential propagates through the atria and into the ventricles in a unidirectional waveform of excitation and relaxation, resulting in a coordinated contraction. The action potential (AP) is generated by the transport of ions through transmembrane ion channels [6], including those of the KCNQ1 (KVLQT1, KV7.1) and hERG (KCNH2, KV11.1) potassium channels and the SCN5A (NaV1.5) sodium channel [7]. Mutations in these three channels are the most common cause of congenital LQTS (cLQTS).

LQTS has been classified into 17 subtypes based on mutations associated with 15 autosomal dominant genes, i.e., LQT1-15 [8]. KCNQ1, hERG, and SCN5A each play a distinct part in generating the cardiac action potential, therefore producing different LQTS forms when mutated. The initial upstroke is regulated primarily by SCN5A, which produces the INa current that amplifies membrane depolarization and propagates the action potential. LQTS type 3 relates to an increased late Na+ inward current (INa, late) resulting from a gain of function in Nav1.5 channels (SCN5A) [9]. hERG forms both the plateau and the repolarization phases of the action potential, concurrently with KCNQ1 in complex with the KCNE1 accessory protein. hERG and the KCNQ1-KCNE1 complex produce rapid (I_Kr_) and slow (I_Ks_) delayed rectifier currents, respectively. KCNQ1 and KCNH2 cause long QT (LQT) type 1 and LQT2, respectively [10]. There are more ion channels and transporters that also form the cardiac action potential, including Cav1.2, NCX1, and Kir2.1; these typically do not cause LQTS.

JLNS is a rare autosomal recessive disorder characterized by a prolonged corrected QT (QTc) interval (usually more than 500 msec) and bilateral sensorineural hearing loss [11]. Homozygous or biallelic compound variants influencing function can lead to JLNS. Moreover, there is an autosomal dominant form, RWS, commonly a clinically milder form with genetically heterozygous missense, nonsense, exon skipping, and frameshift variants [12]. Heterozygous KCNQ1 mutations resulting in a decreased or total loss of function of the Kv7.1 channel cause RWS or type 1 LQTS. Homozygous or compound heterozygous KCNQ1 mutations cause JLNS [13]. KCNQ1 mutations are the most common cause of both of these syndromes.

Clinical features of LQTS

LQTS is a cardiac electrophysiologic disorder, characterized by QT prolongation and T-wave abnormalities on the EKG that are associated with tachyarrhythmias, typically the ventricular tachycardia TdP. TdP is usually self-terminating, thus causing a syncopal event, the most common symptom in individuals with LQTS. Such cardiac events typically occur during exercise and emotional stress, less frequently during sleep, and usually without warning [14]. Clinical manifestations of congenital LQTS include LQTS-attributable syncope, aborted cardiac arrest, and SCD. Many patients with LQTS will remain asymptomatic for life [15]. Sometimes, auditory stimuli, such as a telephone ringing or an alarm clock sounding, will trigger syncopal episodes. A higher incidence of syncope was found to occur during menstruation and in the postpartum period. Fifteen percent of the symptoms occurred during rest or sleep [16].

Diagnosis and role of genetics

**Figure 1:**
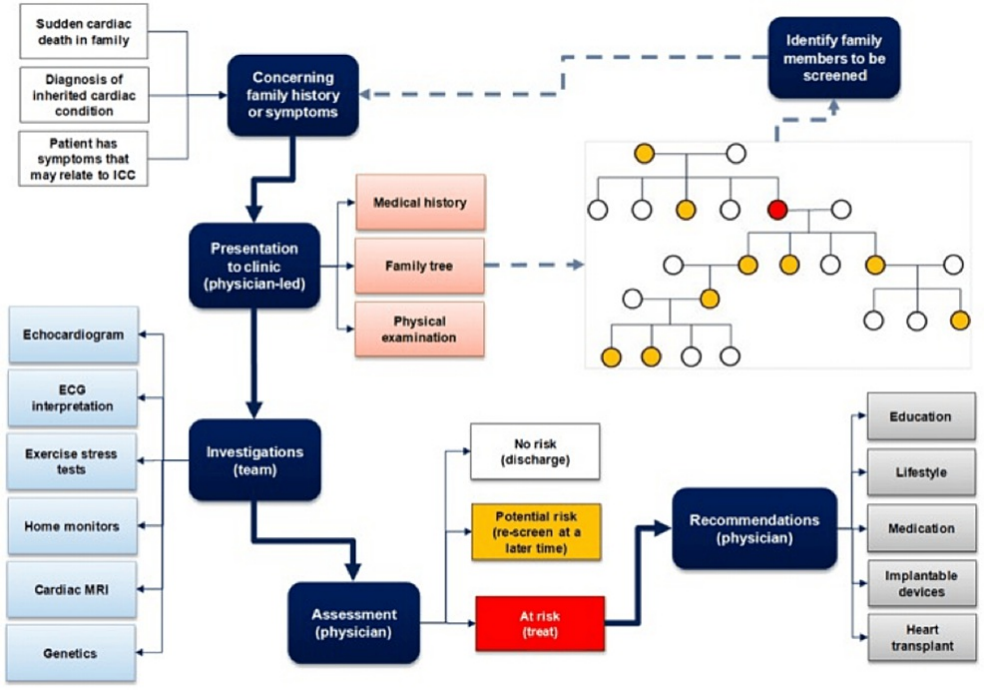
Diagnostic workup of long QT syndrome. Reproduced under the terms of the Creative Commons attribution license [7].

The initial diagnostic evaluation (Figure 1) of LQTS includes obtaining a detailed personal and multi-generation family history, physical examination, series of 12-lead ECG recordings, and calculation of the LQTS diagnostic score, called Schwartz score [15]. From a clinical test standpoint, any patient with a strong clinical index of suspicion for an LQTS diagnosis or an asymptomatic patient with an unequivocal prolonged QTc interval (>480 ms during prepuberty, >500 ms during adulthood) in the absence of other clinical conditions should be offered clinical LQTS genetic testing. However, genetic tests must be understood as probabilistic rather than unconditionally deterministic, and the genetic test results must be interpreted cautiously and incorporated into the overall diagnostic evaluation for these disorders [17]. 

Schwartz score

When a patient satisfies a high-probability Schwartz score (Table 1), i.e., ≥3.5 points, the likelihood of a positive LQTS genetic test is approximately 80%. The intermediate-probability Schwartz score warrants further pursuit of the possibility of congenital LQTS (i.e., genetic testing of the patient and ECG testing of his/her relatives). The likelihood of LQTS is approximately a 5-20% chance, far higher than the one in 2000 background rate for this disease. If the Schwartz score is low (<1 point), genetic testing should not be pursued and these patients should be referred to as normal [15].

**Table 1:** Schwartz score for long QT syndrome. SCD: sudden cardiac death; QTc: corrected QT; low probability ≤1 point; intermediate probability 1.5 to 3 points; high probability ≥3.5 points Reproduced under the terms of the Creative Commons attributions license [15].

ECG findings (in the absence of medications or disorders known to affect these features)
• QTc (= QT/√RR, interpret with caution with tachycardia since QTc overcorrects at fast heart rates)
- ≥480 milliseconds: 3 points
- 460 to 479 milliseconds: 2 points
- 450 to 459 milliseconds (in males): 1 point
• QTc at the fourth minute of recovery from exercise stress test ≥480 milliseconds: 1 point
• Torsades de pointes* (in the absence of drugs known to prolong QT): 2 points
• T-wave alternans: 1 point
• Notched T wave in three leads: 1 point
• Resting heart rate below second percentile for age (restricted to children): 0.5 point
• Clinical findings:
• Syncope* (*points for documented torsades and syncope are mutually exclusive)
- With stress: 2 points
- Without stress: 1 point
• Family history (the same family member cannot be counted in both of these criteria):
• Family members with LQTS: 1 point
• Unexplained SCD in immediate family members <30 years of age: 0.5 point

## Review

Treatment of LQTS

Beta Blockers

Beta blockers are widely used in the treatment of LQTS, a cardiac disorder characterized by prolonged ventricular repolarization. The primary goal of using beta blockers in LQTS is to reduce the risk of life-threatening arrhythmias and sudden cardiac death [18]. The mechanism of action of beta blockers involves blocking the effects of adrenaline (epinephrine) on the heart. By doing so, they slow down the heart rate and decrease the contractility of the heart muscle. This action helps to normalize the prolonged QT interval seen on an ECG. The normalization of the QT interval occurs because beta blockers lengthen the time it takes for the ventricles to repolarize, allowing for a more balanced electrical activity in the heart [18]. By preventing rapid and irregular heart rhythms, beta blockers help to maintain a stable and normal cardiac function. Commonly prescribed beta blockers for LQTS include propranolol, metoprolol, and nadolol. The treatment usually begins with a low dose, which is gradually increased based on individual response and tolerability [19]. Regular monitoring of the ECG and electrolyte levels is important during treatment to assess the effectiveness of therapy and ensure safety. It is important to note that while beta blockers effectively manage LQTS; they are not curative [20]. Therefore, patients should be educated about triggers that can precipitate arrhythmias and advised to avoid or minimize exposure to these triggers. In summary, beta blockers play a critical role in the management of LQTS by reducing the risk of life-threatening arrhythmias and improving overall prognosis [18]. Regular monitoring and patient education are key components of LQTS management.

To examine how sodium channel blockers affect the reduction of QTc and cardiac events [21], the researchers synthesized prior studies (meta-analysis) in the *Journal of Cardiovascular Electrophysiology* investigating relationships between electrophysiological properties and outcomes. They gathered data from 14 studies involving 213 patients for analysis. Results indicated that QTc after using sodium channel blockers was significantly shortened by approximately 50 ms compared to QTc before administration (mean difference (MD), -49.43; 95% confidence interval (CI), -57.80 to -41.05; p<.001) [21]. After analyzing studies, data showed that sodium channel blockers reduced the incidence of cardiac events by nearly 80% (risk ratio (RR), 0.23; 95% CI, 0.11-0.47; p<.001) [21].

Although more than 16 genes have been identified as the cause of LQTS, the most common forms are attributed to mutations in three specific genes: KCNQ1 (LQT1), KCNH2 (LQT2), and SCN5A (LQT3). These mutations account for approximately 75% of clinically confirmed LQTS [22]. To better understand the impacts of sodium channel blockers on QTc in various subtypes of LQTS, this meta-analysis categorized patients into two groups: LQT3 and LQT2. This division was made because there is an insufficient number of patients with LQT1 (n=9) to draw any conclusive findings [21]. The analysis revealed a significant 60 ms (MD, −57.39; 95% CI, −67.41 to −47.37; p<.001) decrease in QTc among LQT3 patients treated with sodium channel blockers. Furthermore, there was a significant reduction in the occurrence of cardiac events by approximately 80% (relative risk (RR) 0.25; 95% CI, 0.12-0.52; p<.001). In addition, there was an approximate decrease of 70% in the proportion of QTc ≥500 (RR, 0.26; 95% CI, 0.17-0.39; p<.001). Notably, there was a substantial increase of 11 times in the proportion of QTc ≤ 460ms (RR, 11.40; 95% CI, 4.73-27.48; p<.001) [21].

Left Cardiac Sympathetic Denervation (LCSD)

Cardiac sympathetic denervation, specifically left cardiac sympathetic denervation (LCSD), is a therapeutic approach used in the treatment of LQTS. LCSD involves surgically interrupting the sympathetic nerve fibers that innervate the heart's left side, which helps to reduce the risk of life-threatening arrhythmias and sudden cardiac death in individuals with LQTS. The rationale behind LCSD is that the sympathetic nervous system plays a role in triggering arrhythmias in LQTS by increasing the heart rate and affecting cardiac repolarization. By selectively interrupting the sympathetic innervation to the heart's left side, LCSD helps to mitigate these triggers and stabilize cardiac electrical activity. LCSD is considered an adjunctive therapy in cases where LQTS remains refractory to conventional treatment, such as beta blockers or implantable cardioverter-defibrillator (ICD) placement [23]. It may be particularly beneficial in patients with recurrent ventricular arrhythmias despite optimal medical management. However, it is important to note that LCSD is an invasive procedure that requires surgical expertise and should be performed by experienced cardiac surgeons. Risks associated with LCSD include surgical complications and potential denervation effects beyond the intended left side. In summary, LCSD is an adjunctive treatment option for patients with LQTS who are unresponsive to conventional therapies. By interrupting sympathetic innervation to the heart's left side, LCSD aims to reduce arrhythmogenic triggers and improve clinical outcomes. Close collaboration between cardiologists and cardiac surgeons is necessary to determine the appropriateness and feasibility of LCSD in individual patients with LQTS [23].

A retrospective review of 52 individuals [24], consisting of 24 males with a mean age at diagnosis of 10.0±10 years and mean QTc of 528±74 ms with diagnosis of LQTS who underwent LCSD (mean age at LCSD, 14.1±10 years) between 2005 and 2010, to determine LQTS-related breakthrough cardiac event (BCE) after LCSD. These patients were followed for 3.6±1.3 years. Out of the total population, 24 individuals (46%) were male, with an average age of diagnosis at 10.0±10 years. Before undergoing the LCSD procedure, the mean baseline QTc was measured at 528±74 ms. Of all the patients, 33 (63%) received LCSD as a primary preventive measure, while the remaining 19 patients (37%) underwent it for secondary prevention purposes. Approximately 63% of the patients were considered to be at high risk for potentially life-threatening arrhythmias. The main reasons for opting for LCSD included intolerance to beta blockers (33%), assessment of severe or high-risk LQTS (25%), experiencing a BCE event while on medication (19%), or simply requiring additional protection measures (23%). Out of the 52 patients who underwent LCSD, 12 patients (23%) experienced at least one BCE event after the procedure. This is a significant decrease compared to the 34 out of 52 patients who had cardiac events before undergoing LCSD (p<0.001). Interestingly, among the 34 patients who previously experienced cardiac events, five patients (15%) did not observe any noticeable reduction in such events after undergoing LCSD. Meanwhile, out of the 29 formerly symptomatic patients, there was a reduction in cardiac events following LCSD. Similar to previous findings, this study observed a reduction in cardiac events in 85% of the patients within the cohort. However, it is important to note that LCSD should not be seen as a cure or an alternative to ICD for high-risk patients who have had an LQTS-related BCE. Among appropriately risk-stratified patients, a prophylactic LCSD, rather than a prophylactic ICD, may represent a robust therapeutic option for patients with unacceptable beta blocker-related side effects [24].

Another study by Niaz et al. [25] concluded that for certain patients who cannot tolerate beta blockers, LCSD may be a viable and effective treatment. However, it is important to note that LCSD alone does not provide a cure for the condition. Therefore, careful patient selection should be a key factor when considering LCSD as a potential monotherapy. A retrospective review of 204 patients with LQTS who underwent LCSD as a standalone monotherapy was performed to assess the effectiveness of LCSD as a standalone treatment option. Out of the 204 patients, 64 (31%) received LCSD as monotherapy. Among these patients, 37 (58%) were female, with an average QTc of 466±30 ms. Prior to diagnosis, 16 patients (25%) experienced symptoms, and their mean age at diagnosis was 17.3±11.8 years. Five patients (8%) had one or more BCEs after diagnosis, and the mean age at LCSD treatment was 21.1±11.4 years. The main reason for choosing LCSD monotherapy was the undesirable side effects of beta-blocker medication that adversely affected the quality of life in 56 out of 64 patients (88%). The underlying LQTS genotype was LQT1 in 36 cases (56%) and LQT2 in 20 cases (31%). There were no significant complications related to LCSD surgery. During a mean follow-up period of approximately 2.7±2.4 years thus far, only three patients experienced non-life-threatening post-LCSD BCEs within a span of 180 patient years [25].

ICD Implantation

ICD placement is a well-established therapeutic option for the treatment of LQTS. It is primarily recommended for individuals with LQTS who are at a high risk of life-threatening arrhythmias and sudden cardiac death [23]. ICDs are small electronic devices that are surgically implanted under the skin, typically in the chest area. They continuously monitor the heart's electrical activity and deliver electrical shocks or pacing therapy when abnormal rhythms are detected. In the case of LQTS, ICDs are specifically programmed to detect and treat ventricular tachyarrhythmias, which are the primary cause of sudden cardiac death in LQTS patients. The decision to implant an ICD in patients with LQTS is based on various factors, including the severity of the condition, presence of known risk factors for arrhythmias, and previous history of cardiac events. It is particularly considered in individuals who have experienced syncopal episodes or cardiac arrests despite optimal medical therapy with beta blockers [26]. While ICDs are highly effective in terminating life-threatening arrhythmias, it is essential to carefully consider the potential risks and benefits associated with their placement. ICD implantation carries procedural risks, such as infection, lead-related complications, and the need for future device revisions or replacements. Close follow-up and programming adjustments are necessary after ICD implantation to ensure optimal device function and patient safety. In addition, patient education regarding appropriate responses to ICD therapies, lifestyle modifications, and ongoing management of LQTS is crucial. In summary, ICD placement is an important treatment option for patients with LQTS at high risk of life-threatening arrhythmias [26]. It provides continuous monitoring and delivers appropriate therapies to terminate potentially fatal ventricular arrhythmias. The decision to implant an ICD should be made based on individual patient factors, and close follow-up is necessary for device management and patient education.

A study comparing the in-hospital outcomes of LCSD and ICD in the management of LQTS analyzed a total of 9,365 cases of LQTS [27]. Among them, 5,945 cases received an ICD, while 3,420 cases underwent treatment with LCSD. The ICD cohort was relatively younger with an average age of 58 years compared to the LCSD cohort with an average age of 65 years. It was observed that the ICD cohort had seven times higher in-hospital mortality rates than the LCSD cohort (0.59% vs. 0.084%; p=0.04). When comparing LCSD and ICD, it was found that ICD had significantly higher odds of various cardiac events. Specifically, the adjusted odds ratio (aOR) for cardiac arrest was 21.4, myocardial infarction was 6.31, cardiogenic shock was 1.67, and mechanical ventilation was 7.45 (p<0.05) [27].

Gene-Specific Therapy

Gene-specific therapy is an emerging approach in the treatment of LQTS that aims to target the underlying genetic mutations responsible for the condition [23]. It involves the use of specific medications or interventions that directly address the genetic defect, with the goal of correcting or mitigating its effects on cardiac ion channels and repolarization. Gene-specific therapy is primarily considered in cases of LQTS where the specific genetic mutation has been identified and its functional consequences are well understood. Different strategies are being explored, including the use of pharmacological agents that modulate ion channel function or gene-editing techniques that aim to correct the genetic defect itself [23]. For example, in cases of LQTS caused by mutations in the KCNQ1 gene (LQTS type 1), drugs, such as mexiletine and ezogabine, have shown promise in enhancing channel function and restoring normal repolarization. Similarly, gene-editing techniques, such as CRISPR/Cas9, hold the potential for correcting specific genetic mutations responsible for LQTS. While gene-specific therapy is a promising avenue for personalized treatment, it is still in the early stages of development and research. Further studies are needed to assess the safety, efficacy, and long-term outcomes of these approaches. In addition, the availability and feasibility of gene-specific therapy may vary depending on the specific genetic mutation involved. In summary, gene-specific therapy represents an exciting area of research for the treatment of LQTS. It aims to directly target the underlying genetic mutations responsible for the condition and holds the potential for personalized and precise interventions. However, further investigation is needed to fully understand its safety, efficacy, and clinical applicability. Collaboration between cardiologists, geneticists, and researchers is crucial in advancing the field of gene-specific therapy for LQTS.

A retrospective cohort study [28] examined 34 patients diagnosed with LQT3 who were treated with mexiletine. The goal was to assess the effectiveness of mexiletine as an antiarrhythmic drug by comparing the number and rate of arrhythmic events per patient before and after starting treatment. Of 34 patients diagnosed with LQT3, 19 were males (56%). The average age at the start of mexiletine treatment was 22 years, and the initial QTc interval was measured at 509 ms. On average, patients took oral mexiletine for a duration of 36 months, with a daily dose averaging 8±0.5 mg/kg. Treatment with mexiletine resulted in significant improvements, including a reduction in QTc interval by an average of 63±6 ms (p<0.0001). The use of mexiletine also led to a decrease in the percentage of patients experiencing arrhythmic events from 22% to 3% (p<0.031) and a decrease in the mean number of arrhythmic events per patient from an average of 0.43±0.17 to 0.03±0.03 (p=0.027). In addition, there was a substantial decline in the annual rate of arrhythmic events from 10.3% to 0.7%; p = 0.0097. These data corroborate that mexiletine may improve survival in patients with LQT3 [28].

Ezogabine is a recently developed medication used to treat epilepsy. Its mechanism of action involves modifying potassium channels in the brain. Specifically, ezogabine targets neuronal potassium channels (KCNQ2 and KCNQ3), prolonging their time open and delaying the process of membrane repolarization. These channels are encoded by a family of genes that regulate cellular excitability by influencing the M current. One particular channel, KCNQ1, is primarily expressed in the heart, and dysfunction can cause LQTS. Meanwhile, KCNQ2 and KCNQ3 channels are found in the brain, and deficiencies in these may lead to benign familial neonatal convulsions. Ezogabine's anticonvulsant effects stem from its selectivity for KCNQ2 and KCNQ3 channels as positive allosteric modulators. By enhancing these channels' activity, it helps decrease neuronal hyperexcitability. It is worth noting that ezogabine does not have any significant effect on cardiac cells with regard to KCNQ1. Despite this specificity in action, there have been rare instances of modest QT prolongation observed during phase III clinical trials of ezogabine. In healthy subjects who were administered up to 1200 mg/day of the drug, an average increase in QT interval duration of 7.7 ms was reported [29].

Polypharmacy and LQTS

Polypharmacy is the simultaneous use of multiple medications in a single individual. There is a large heterogeneity in the definition of polypharmacy, ranging from numerical counts only, numerical counts for a given duration of therapy or setting, or descriptive, which includes terms, such as minor, moderate, major, and excessive polypharmacy [30]. Based on the review of current data, the use of five or more medications is an acceptable definition of polypharmacy [31]. It has been postulated that while the term polypharmacy has evolved over time, the basis for the definition is simply more drugs being prescribed or taken than are clinically appropriate in the context of a patient’s comorbidities [30].

**Figure 2:**
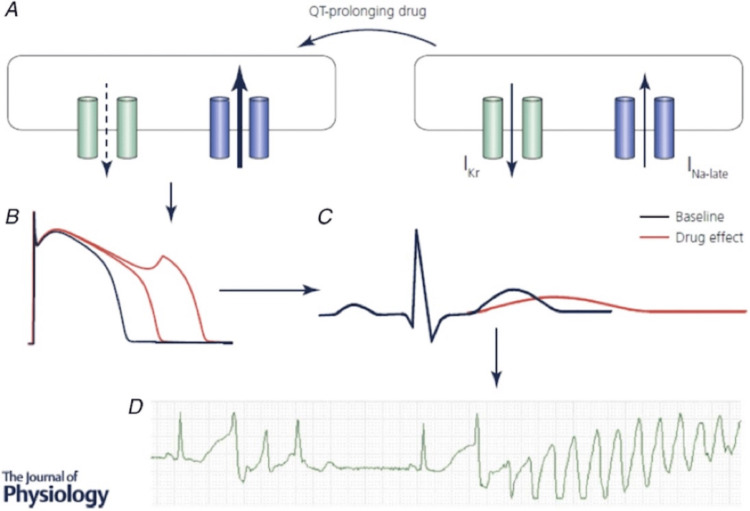
Pathophysiology leading to QT interval prolongation. Reproduced under the terms of the Creative Commons attribution license [34].

In a prospective study of intensive care unit (ICU) patients with continuous QT monitoring, 24% of the patients had QT interval prolongation (defined as QTc>500 ms for ≥15 min) during ICU stay, and 6% of in-hospital cardiac arrests were due to TdP arrhythmia [32]. ICU patients with QT prolongation have higher hospital mortality (8.7% vs. 2.6%; p<0.0005) compared with ICU patients without QT prolongation [32]. The most common acquired cause of LQTS is drug-induced QT interval prolongation [33]. Therefore, polypharmacy including QT-prolonging drugs has been shown to worsen LQTS. These drugs mainly act by interfering with the rectifying potassium current, which is responsible for the repolarization of the cardiac myocytes. This causes slow repolarization, which increases the interval from the start of ventricular cardiac myocyte depolarization to the end of repolarization, corresponding to the QT interval on the ECG (Figure 2).

QT-prolonging drugs are shown in Table 2.

**Table 2:** Drugs prolonging the QT interval.

Drug class	Drugs	Mechanism of action	Notes
Anesthetics	Propofol, sevoflurane	Inhibition of I_Ks_ (slow component of delayed rectifier potassium current) and I_to_ channels (transient outward potassium current) [35]	
Antiarrhythmics	Amiodarone, sotalol, quinidine, procainamide, verapamil, and diltiazem [33]	Extended duration of ventricular repolarization leading to a prolonged QT interval	The incidence of drug-induced TdP with intravenous amiodarone is about 1.5% [36].
Antibiotics	Azithromycin, ciprofloxacin, clarithromycin, erythromycin, levofloxacin, moxifloxacin, and roxithromycin	Inhibition of I_Kr_ (a rapid component of delayed rectifier potassium current) [35]	
Anticancer	Aclarubicin, arsenic trioxide, oxaliplatin, and vandetanib	Arsenic trioxide works by inhibition of I_Kr_ trafficking, while vandetinib works by I_Kr_ inhibition [35].	
Antidepressants (selective serotonin reuptake inhibitors)	Citalopram and escitalopram	Inhibition of both I_Kr_ and I_Kr_ trafficking [35]	
Antiemetics	Domperidone, droperidol, and ondasetron	Inhibition of I_Kr_ [35]	
Antifungals	Fluconazole and pentamidine	Fluconazole acts by I_Kr_ inhibition and inhibition of I_Kr_ trafficking, while pentamidine works by inhibition of I_Kr_ trafficking [35].	
Antimalarial drugs	Chloroquine, hydroxychloroquine, and halofantrine	Inhibition of I_Kr_ [35]	The overall incidence of QT interval prolongation associated with hydroxychloroquine or chloroquine is ≈10%, although the incidence increases when azithromycin is added to therapy [35].
Other drugs	Donepezil, papaverine HCL, cocaine, terodiline, methadone, anagrelide, cilostazol, cesium chloride, and terlipressin	Inhibition of I_Kr_ [35]	

Antipsychotics and their link to LQTS

Antipsychotic agents are a class of drugs that are utilized in the treatment of psychiatric conditions, including schizophrenia and bipolar disorder. Their mechanism of action involves altering the activity of neurotransmitters in the brain, which aids in relieving symptoms commonly associated with psychosis [37]. Some psychotropic medications have been linked to a potentially life-threatening condition called TdP, which is characterized by lethal ventricular arrhythmias. This association is particularly prevalent in individuals with medical illnesses. The mechanism behind the QT interval prolongation caused by these medications is believed to involve their impact on potassium channels and the hERG gene. By affecting the duration of potassium channel opening, these medications can lead to prolonged ventricular repolarization and subsequently an extended QT interval. It has been observed that up to 90% of patients who experience TdP while using non-antiarrhythmic medication, including psychotropic drugs, have a QTc value exceeding 500 milliseconds [38].

The QT interval, which is the time between the start of the Q wave and the end of the T wave on an ECG, measures ventricular depolarization and repolarization. To account for heart rate variation, it is more meaningful to use QTc. Several formulas, such as Bazett's formula and Fridericia's formula, can calculate QTc. The normal values differ between males and females, with a prolonged QTc defined as exceeding 450 ms in males and 470 ms in females [38]. Select antipsychotic prescriptions have proven connections to either instigating the onset of LQTS or prompting TdP episodes. The means by which these antipsychotic agents can result in LQTS relates to their influence on the ion pathways within the cells of the heart [34]. The cardiac cells' activity possibility depends on the orderly unfastening and fastening of assorted ion passages. The length of the activity possibility is mirrored in the QT interim on the ECG. Expansion of the QT interim elevates the threat of irregular heartbeats, particularly TdP [34].

The majority of patients requiring psychotropic medications generally have few risk factors for QTc prolongation. Consequently, they can be considered at low risk for TdP. The frequency of cardiac monitoring necessary for patients receiving psychiatric medications should be determined on an individual basis, taking into account the specific prescribed agent(s) and any additional risk factors related to TdP [39].

Psychopharmacology

Sertindole emerged as the molecule most prone to correlate with accounts of this cardiac irregularity, trailed in a decreasing order of probability by the substances ziprasidone and amisulpride. By contrast, lurasidone exhibited the smallest risk [40]. The initial generation of antipsychotics was established to report a hazard ratio of 1.21 (95% CI, 1.10-1.33) for QT prolongation compared with the newer atypical antipsychotics [40]. A direct relationship was detected between the risk of QT prolongation accounts and affinity for the hERG-related gene ion channel (R^2^=0.14, slope of the line=Pearson coefficient=0.41; p-value=0.1945) [40]. This expansive examination in an authentic milieu shows that sertindole and ziprasidone were the antipsychotic medications affiliated with the maximal jeopardy of QT extension announcing. These conclusions propose that lurasidone is less connected with QT interval extension reports. This research likewise proposes that antipsychotics with superior hERG affinity are more associated with QT prolongation reports [40].

Pharmaceuticals provoke irregular heart rhythm primarily by impeding the ion channels within cardiac cells. These channels, permeable portals facilitating the flow of charged atoms, are obstructed by chemicals. The rhythm of cardiac muscle depends on the synchronized opening and closing of these channels, allowing for the controlled passage of ions at precise moments. When inhibited, the orchestrated beat is disrupted, resulting in a chaotic, dysfunctional cadence [35]. The cardiac myocyte's delayed rectifier potassium current experiences impedance, prompting the prolonging of the cardiac cell's excitation period. This hindrance manifests in the myocyte's repolarization phase, forestalling the return of the cell's membrane potential to a restful state. Specifically, the rapid component of the delayed rectifier potassium current encounters restraint. Consequently, the rapid delayed rectifier's capability to expedite repolarization and abbreviate the action potential is fettered [35].

The gaping susceptibility to premature heartbeats subsists. Anomalous heart rhythms can arise prior to the refractory period. The lingering impact of a recent heartbeat may trigger another to commence too rapidly. These unanticipated heartbeats transpire earlier than anticipated and disrupt the orderly sequence of heartbeats. The timing and source of these premature beats can vary. The capacious cardiac cells can catalyze an arrhythmia through the secondary ingress of an electrical impulse. Potassium channels in myocardial tissue occasionally reopen, enabling another depolarization to develop that spreads as a reentrant circuit. This abnormal cardiac rhythm, known as TdP, can emerge from a prolonged QT interval succeeding the initial activation of cardiac muscle [35]. The chemicals primarily disturb the rapid depolarization of the cardiac muscle fibers through the impediment of the potassium ion channels. This disruption culminates in a lengthening of the QT interval as gauged on the ECG. In certain individuals with a predisposition toward prolonged repolarization, the drugs may facilitate the onset of the lethal arrhythmia TdP [41].

The abnormality of the cardiac transient outward potassium current, or I_Kr_, culminates in an elongated period of the heart's pumping potential. An abnormal heartbeat rhythm known as TdP may be induced by allowing electrical impulses in the heart to re-enter their second phase, thereby decreasing the likelihood of premature contractions [41]. The cardiac cell's electrical restructuring period encompasses a distinct physical event that may arise within heart muscles during the recalibration interval of the activation possibility. Corkscrew apexes de markers (corkscrew apexes) embody a distinct ventricular tachycardia type denoted by a singular ECG design with twisting apexes and can possibly be activated by the cardiac cells' electrical restructuring period [41].

Certain pharmaceuticals containing dofetilide, ibutilide, and d-sotalol, aim to rectify heart arrhythmias. These antiarrhythmic agents endeavor to reinstate usual heart rhythms by impeding erratic electrical impulses in the organ. However, these compounds can also provoke hazardous heart rhythms in some patients. Patients who are administered these drugs require careful monitoring to rapidly detect irregular heartbeats. The psychotropic substance, along with the antibiotic, additionally extends the duration of the lower heart chambers' systole [41]. Succession of action potential resulted in part through the amplification of an inward current. This inward flow, elicited by the opening of ion channels, caused a positive feedback mechanism whereby the membrane potential became less negative. The cycle repeated itself; with each iteration, the inward current increased and the membrane potential rose. Once the membrane potential reached a critical threshold, the voltage-gated sodium channels deactivated and an outward flow of potassium ions repolarized the membrane [41].

A modern influx of pharmaceutical innovators have developed compounds targeting the enduring sodium stream (INa-L); a few of these creations, despite affecting the cardiac muscle's ability to rapidly depolarize, nonetheless left the heart's pumping power unscathed [41]. The consequence arises from influences on the lipid messenger. The lipid messenger then impacts the enzyme kinase, which phosphorylates the phosphoinositide. This phosphoinositide phosphorylation leads to the activation of Akt, a serine/threonine kinase. Akt stimulates cell growth and proliferation through various downstream effects. One of these effects is the activation of the mammalian target of rapamycin (mTOR), which then stimulates protein synthesis and cell growth [41]. The heart's electrical cycle comprises discrete periods: commencement (stage 0), premature reversion (stage 1), standstill (stage 2), and restoration (stage 3) [41]. The heart cell membrane endures an extended duration of limited repolarization as calcium flows inward to counterbalance potassium currents outward throughout the plateau period (stage 2). This interval sustains the plateau and provides for unified compression of the heart [41].

Below certain conditions, such as particular electrolyte disturbances or select drugs, the equilibrium of ion streams during this second period could be disrupted. This interference might lead to an irregular electrical event, termed early afterdepolarizations (EADs). EADs constitute abnormal depolarizations transpiring during the plateau interval rather than the anticipated repolarization [41]. An aberrant conduction can induce an untimely systole if arriving at a crucial limit, prompting an unscheduled systole. This unscheduled systole may re-enter the cardiac muscle through a winding path, forming a re-entry cycle. As this irregular electrical wavefront spreads through the ventricles, it is capable of producing a warped model of the QRS composite on the EKG, emblematic of TdP [41]. The reappearance of state 2 arrival and resulting commencement of TdP relates to particular influences, encompassing an elongated QT period (a gauge of repolarization extent), electrolyte irregularities (e.g., potassium deficiency or magnesium inadequacy), and specific treatments influencing ion channel performance (e.g., some antiarrhythmic compounds) [41].

The interconnectedness between the second stage of re-entry and a dangerous heart rhythm is intricate. The precise processes that underlie this precarious heart rhythm can differ contingent upon the explicit fundamental circumstance or catalyzing influences. Those in the medical field thoroughly observe individuals with risk factors for this precarious heart rhythm and take suitable actions to govern and deter its occurrence [41]. The risk of drug-induced prolonged QT interval and TdP has been described with the repolarization reserve concept, which postulates that repolarization is modulated by multiple partially redundant mechanisms, including the balance of function of I_Kr_, the slow component of the delayed rectifier potassium current (I_Ks_), INa-L, and other ion currents. The repolarization reserve theory posits that when one determinant of repolarization is abnormal or inhibited, repolarization (and the QT interval) may remain normal or close to normal. However, when an additional perturbation occurs, such as the introduction of an I_Kr_-inhibiting drug, hypokalemia, hypomagnesemia, or other negative repolarization influences, the repolarization reserve becomes diminished, resulting in QT prolongation and increasing the risk of TdP [34]. Table 3 shows the therapeutic drug classes associated with TdP.

**Table 3:** Therapeutic drugs leading to torsades de pointes. Reproduced under the terms of the Creative Commons attribution license [34].

Therapeutic class	Examples of "high-risk" drugs
Antiarrhythmics	Dofetilide
	Ibutilide
	Quinidine
	Sotalol
Antibiotics	Grepafloxacin**
	Pentamidine
	Sparfloxacin**
Anticancer agents	Arsenic trioxide
	Vandetanib
Antiemetics	Droperidol
	Ondansetron
Antihistamines	Astemizole**
	Terfenadine**
Antipsychotics	Haloperidol
	Mesoridazine**
	Pimozide
	Thioridazine
Gastric prokinetic agents	Cisapride**
Opiate antagonists	Levomethadyl**
	Methadone
*Not all members of a presented class share equal liability; generally, one or more drugs have especially high liability, and others have no liability. Specific lists are maintained at www.crediblemeds.org. **Withdrawn from the US market because of torsades de pointes liability.	

**Figure 3:**
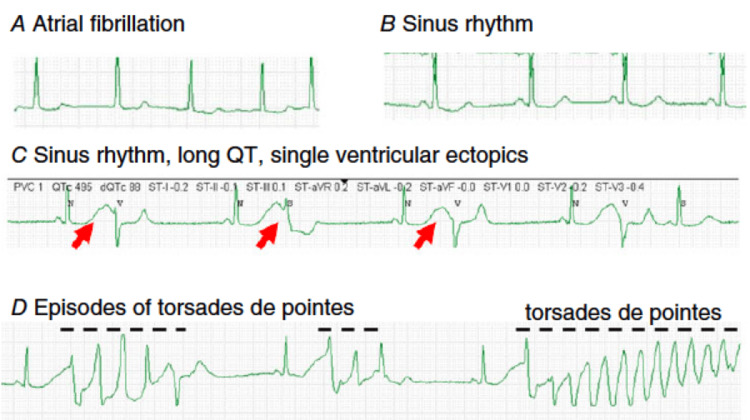
Development of torsades de pointes in a patient with atrial fibrillation treated with sotalol. A: Atrial fibrillation. B: With sotalol, the rhythm converts to sinus with a borderline prolonged QT interval (480 ms). C: Several hours later, the QT interval after sinus beats is markedly prolonged (>600 ms; red arrows), and single ectopic beats, possibly representing early afterdepolarization-mediated automaticity (Abstract Figure B) are seen. D: This is followed within minutes by bursts of the polymorphic ventricular tachycardia torsades de pointes, indicated by the dashed lines. Reproduced under the terms of the Creative Commons attribution license [34].

Development of TdP in a patient with atrial fibrillation treated with sotalol is shown in Figure 3.

Atypical antipsychotics and LQTS

For many years, antipsychotic medications have been a crucial treatment option for schizophrenia. These medications work by binding to dopamine receptors in the brain, effectively regulating the symptoms of the disorder. Antipsychotic medications are categorized as first-generation antipsychotics (FGAs) or typical antipsychotics and second-generation antipsychotics (SGAs) or atypical antipsychotics. These medications have variations in their pharmacokinetics and receptor affinity, which leads to different side effects. Traditional or typical antipsychotics can cause side effects, such as extrapyramidal signs and symptoms (EPS) and an increase in serum prolactin levels. Notably, atypical antipsychotics, when used within appropriate dosage ranges, are known to reduce the occurrence of extrapyramidal symptoms [42]. Patients with mental health disorders have been found to have higher mortality rates. In particular, the use of antipsychotic medications carries a risk that is more than twice as high for experiencing sudden death. This risk is believed to be linked to the prolongation of the QT interval and the increased likelihood of developing TdP [43]. This risk occurs because the inward rectifying potassium current is inhibited. As a result, the repolarization of cardiac myocytes is prolonged, leading to an extended QT interval on an ECG. Prolonged QT intervals are associated with a higher risk of TdP and sudden cardiac death [43].

There was no significant QTc prolongation observed with lurasidone, aripiprazole, paliperidone, and asenapine. However, there are additional clinical risk factors that contribute to the potential for drug-induced TdP. These factors include the inherent risk associated with the drug itself, higher doses, rapid titration or infusion, female sex, electrolyte imbalances (e.g., hypokalemia and hypomagnesemia), bradycardia, congestive heart failure (CHF), concomitant use of other medications with QT-prolonging effects, ion-channel polymorphisms, and those with cLQTS caused by ion channel mutations. For patients with risk factors, it is reasonable to get an initial ECG to evaluate the duration of the QT interval [43]. Cardiac myocytes have a naturally negative charge at rest. During an action potential, sodium-dependent inward currents dominate, leading to depolarization. This is followed by a combination of inward sodium and calcium currents and then outward potassium-dependent currents, causing repolarization. When there is an imbalance in the flow of ions across the cell membrane, particularly in potassium current impairment, it can lead to longer repolarization time, resulting in a prolonged QT interval [43]. When it comes to repolarization, two types of potassium rectifier currents are involved: I_Kr_ (rapid rectifier) and I_Ks_ (slow rectifier). Out of these two, the I_Kr_ current is particularly susceptible to pharmacological impact. Inhibiting the I_Kr_ current is primarily responsible for lengthening the repolarization process and consequently prolonging the QT interval [43].

Individual medications that had statistically significant increased QR included FGAs clothiapine, haloperidol, prochlorperazine, and thioridazine, while the SGAs or atypical antipsychotics included quetiapine, risperidone, and sulpiride. They also showed that on average, those drugs with higher potassium channel blockade correlated to a high risk of significant cardiovascular disease [44]. Blocking potassium currents other than I_Kr_ may have proarrhythmic effects, but the impact on other ion channels might counteract this tendency. Some researchers suggest that the simultaneous blocking of calcium and potassium channels could potentially decrease the risk of proarrhythmia associated with QTc prolongation [45]. Atypical antipsychotic medications, such as quetiapine, remoxipride, clozapine, olanzapine, sertindole, ziprasidone, and amisulpride, bind less tightly than dopamine to the dopamine D2 receptor. Their dissociation constants are higher for dopamine compared to typical antipsychotic medications [42].

Atypical Antipsychotics

Sertindole was first introduced to the market in 1997. However, it has been observed that there is a potential for dose-dependent QTc prolongation, with an average increase of 21 msec at the maximum recommended dosage of 24 mg/day [46]. Various reports indicate that the proportion of patients experiencing QTc values ≥500 msec ranges from 3.1% to 4.0% [47], and one document even cites this value as high as 7.8% [48]. In a randomized trial comparing sertindole to haloperidol, it was found that 8% of patients on sertindole had QTc intervals ≥500 msec, while none in the haloperidol group exhibited this (with p<0.05) [49]. Among various antipsychotic medications, a study found that ziprasidone had a more pronounced effect on the QTc interval compared to risperidone, olanzapine, quetiapine, and conventional antipsychotics [48]. Comparing ziprasidone with sertindole is complex because it requires analyzing data from different studies. However, preliminary comparisons suggest that ziprasidone has a less significant impact on repolarization. In short-term trials, ECGs obtained at random intervals showed a mean increase in QTc (Bazett) of 9.7 msec at the highest approved dose of 160 mg/day for ziprasidone [48].

QTc prolongation of ≥500 msec is relatively rare with ziprasidone. Effects on QTc prolongation with ziprasidone are less than those observed with thioridazine [48] and sertindole [46]. In conclusion, the potential of ziprasidone to prolong the QTc interval raises concerns about its risk of causing TdP and sudden death. However, further evaluation through extensive clinical use and postmarketing surveillance is necessary to ascertain its actual impact [45]. Among atypical antipsychotics, risperidone has a significantly lesser impact on the QTc interval compared to sertindole and ziprasidone. Although risperidone can cause minor QTc prolongation at recommended doses, studies have also shown this effect in cases of overdose. It is worth noting that there have been no reported instances of TdP in patients prescribed with risperidone [45]. Unlike sertindole and ziprasidone, other atypical antipsychotics, risperidone has a significantly smaller impact on the QTc interval. While therapeutic dosages of risperidone have been shown to cause minor QTc prolongation, this effect has also been observed in cases of overdose. There have not been any reported instances of TdP in patients prescribed with risperidone [50].

Clozapine has been found to have a dose-dependent effect on increasing QTc intervals, but it rarely reaches pathological levels [51]. Out of the 61 patients who received clozapine in a research study, two patients had a QTc measurement above 500 ms. One of these patients already had an abnormal baseline ECG, and the other patient's QTc value returned to normal even though their clozapine dosage was increased. Therefore, it is unclear whether clozapine played a significant role in affecting the QTc interval or not [50]. In a thorough examination of six different clinical trials, which included a total of 537 patients, zotepine resulted in a modest but significant extension of the QTc interval. The average increase measured at 8.3 msec. Out of the patients who received zotepine treatment, approximately 20% (106 individuals) experienced a QTc prolongation exceeding 30 msec, and around 4% (21 individuals) encountered a QTc prolongation surpassing 60 msec [52].

By contrast, only 9% of the participants who received a placebo experienced a QTc prolongation greater than 30 msec, and none had a QTc prolongation greater than 60 msec. The product monograph states that zotepine has a mild impact on the QTc interval, which is unlikely to have a significant pro-arrhythmic effect [52]. When choosing an antipsychotic, it is important to take several factors into account, including QTc prolongation. Each patient should be considered individually when making this decision. However, because the effectiveness of antipsychotics is generally similar and QTc prolongation can lead to serious consequences, it is wise to avoid medications that have a significant impact on the QT interval whenever possible [45].

Ethnicity and race in LQTS

LQTS is an autosomal dominant hereditary condition that affects the heart's electrical activity. It is estimated to occur in about one in 5000 individuals [53]. The relationship between QT interval and mortality is significantly influenced by ethnicity, as evidenced by studies, such as the Atherosclerosis Risk In Communities (ARIC) study and the Duke Databank, which have shown a stronger association between QT prolongation and cardiovascular mortality in the Black population compared to the White population [54,55]. In the LQTS Registry, African-Americans were found to have a significantly longer QTc interval than Caucasians. However, after adjusting for relevant factors, both groups exhibited similar risks for cardiac events [56]. Studies conducted on healthy populations have revealed that African-American men and women, aged 25 to 74, tend to have QTc intervals that are, on average, 2-5 ms shorter than those of Caucasians [57]. It is worth noting that African Americans have a higher prevalence of hypertension, a leading cause of left ventricular hypertrophy, which in turn can contribute to a prolonged QT interval [58,59].

A 2016 study [60] discovered that among Asians, there were distinct differences in the QT interval based on gender. Asian men had shorter QT intervals compared to White men, while Asian women had longer QT intervals compared to White women. These discrepancies in ECG characteristics were not accounted for by known risk factors for cardiovascular disease. The study also revealed that the impact of certain ECG characteristics on prognosis varied based on race. Specifically, Asians with left ventricular hypertrophy had a significantly higher risk of death compared to the White population. This study emphasized the contrasting race-based differences in QT intervals, with Asian men having shorter QT intervals and Asian women having longer QT intervals compared to their White counterparts. Notably, the gender disparity in the QT interval was even more prominent among Asians. The exact underlying mechanism for these differences is not fully understood, but it is speculated that sex hormones may influence myocyte conduction. Factors, such as diet, environment, and genetic variations, have been linked to differences in testosterone production and metabolism between Asians and the White population, potentially contributing to the observed variations in QT interval distribution among these populations [61-66].

While the QT interval did not show differential predictive patterns for prognosis among races, there were notable sex differences. A longer QTc interval was associated with a worse prognosis in men but not in women [60]. In addition, the study highlighted that racial susceptibility played a more significant role than the class of drugs in determining the risk of QT prolongation following acute drug overdose. The Black population had over twice the increased risk, whereas Hispanics exhibited a reduction of approximately 50% in the risk of drug-induced QT prolongation. Furthermore, differences in ECG findings between racial groups have been documented independently of risk factors for coronary heart disease. For instance, in a racially mixed population of apparently healthy individuals, African-Americans had shorter QTc intervals compared to the White population, even after considering conventional risk factors for coronary heart disease. These findings indicate that racial disparities in ECGs cannot be solely explained by established risk factors, highlighting the importance of considering race when interpreting ECG results [67-69].

Gender differences in LQTS

LQTS comprises categories of cardiac electrophysiologic disorders linked with the dysfunction of the cardiac conducting system resulting in the prolongation of the QT interval and T-wave abnormalities on the ECG [70]. The QT interval represents the depolarization of a cellular membrane [70]. The proportion of LQTS in the general population is about 0.05%, with a slight female preponderance [2]. The biological male differs from the female in the electrophysiological functioning of their specialized conduction system and myocardium, which increases the tendency of the female to develop LQTS [71].

**Figure 4:**
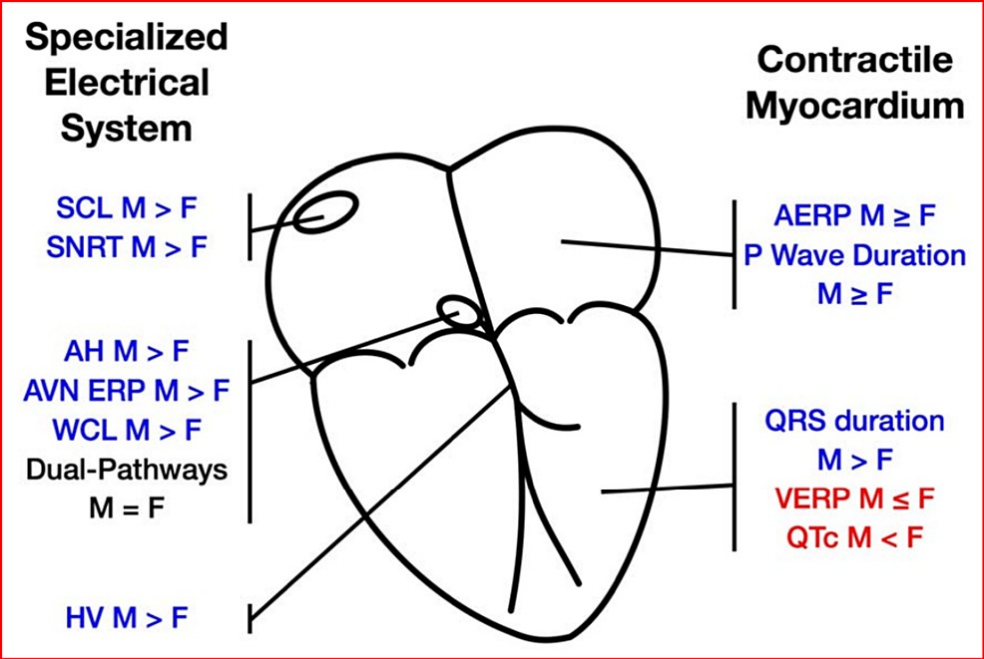
Cardiac conduction system. AH, HV=AH, HV intervals; AERP: atrial ERP; AVN: AV node; ERP: effective refractory period; F: females; M: males; SNCL: sinus-node cycle length; SNRT: sinus-node recovery time; VRP: ventricular ERP; WCL: Wenckebach cycle length Reproduced under the terms of the Creative Commons attributions license [72].

The sinus node rhythm cycle is highlighted as follows [71]: The PA interval is the time for activation to travel into the right atrium between the region of the sinus node and the atrioventricular node (AVN). It is measured between the onset of the P wave and the atrial electrogram recorded by the His bundle catheter. The standard value is 25 to 55 ms. The AH interval is the duration of depolarization over the AVN and is measured between the atrial electrogram recorded on the His bundle catheter and His electrogram. The standard value is 55 to 125 ms. H is the duration from the last component of the His bundle electrogram. The standard value is less than 30 ms. HV is the conduction time over the ventricular conduction system and is measured between the His bundle electrogram and the earliest ventricular activation. The standard value is 35 to 55 ms [71]. Figure 4 shows the cardiac conduction system.

Sinoatrial node (SAN)/AVN/His-Purkinje fiber electrophysiology

The heart's primary pacemaker is the sinoatrial node (SAN), which generates electricity for depolarization because of its automaticity. The SAN functions slightly differently for females because of their higher resting and intrinsic heart rates and shorter corrected SAN recovery time than males who have a slower resting and intrinsic heart rate due to their longer corrected SAN recovery time and atrial refractory period [72]. This shorter cardiac sinus cycle length makes the female’s heart generate impulses faster than the male’s. Although the SAN responds differently for both sexes after the autonomic blockade, the heart rate during exercise depends on individual capacity rather than intrinsic sex differences. The delay in the AVN is shorter for women as evidenced by shorter PR and AH intervals, AV nodal effective refractory period, Wenkebach cycle length, HV interval, and QRS duration. The mechanism of the differences in response by both sexes in AV conduction is unknown [72].

A few mechanisms that have been suggested in the proneness of the female gender to LQTS are estrogen-mediated reduced repolarization reserve in women, androgens, 17B estradiol, and molecular targets, such as inward rectifier potassium current (1K1) [71]. In the atrium, males have longer P-wave duration and longer refractory period, while in the ventricles, the females have longer QT and QTc intervals independent of autonomic modulation. Hormonal-related changes that cause different responses by gender are not observed in prepubertal children. At puberty, males show short QTc while females maintain a slight increase in their QTc. The QT interval is determined by ventricular muscle action potential duration (APD). Longer APDs could result from smaller outward K^+^ currents or larger inward Na+ or Ca^2+ ^currents. Low-dose testosterone has been suggested to reduce APD via enhancement of I_Ks_, whereas high-dose testosterone shortens APD by increasing I_Ks_ and reducing larger inward L-type Ca^2+^ current (I_CaL_) and a reduced QT interval [72].

**Figure 5:**
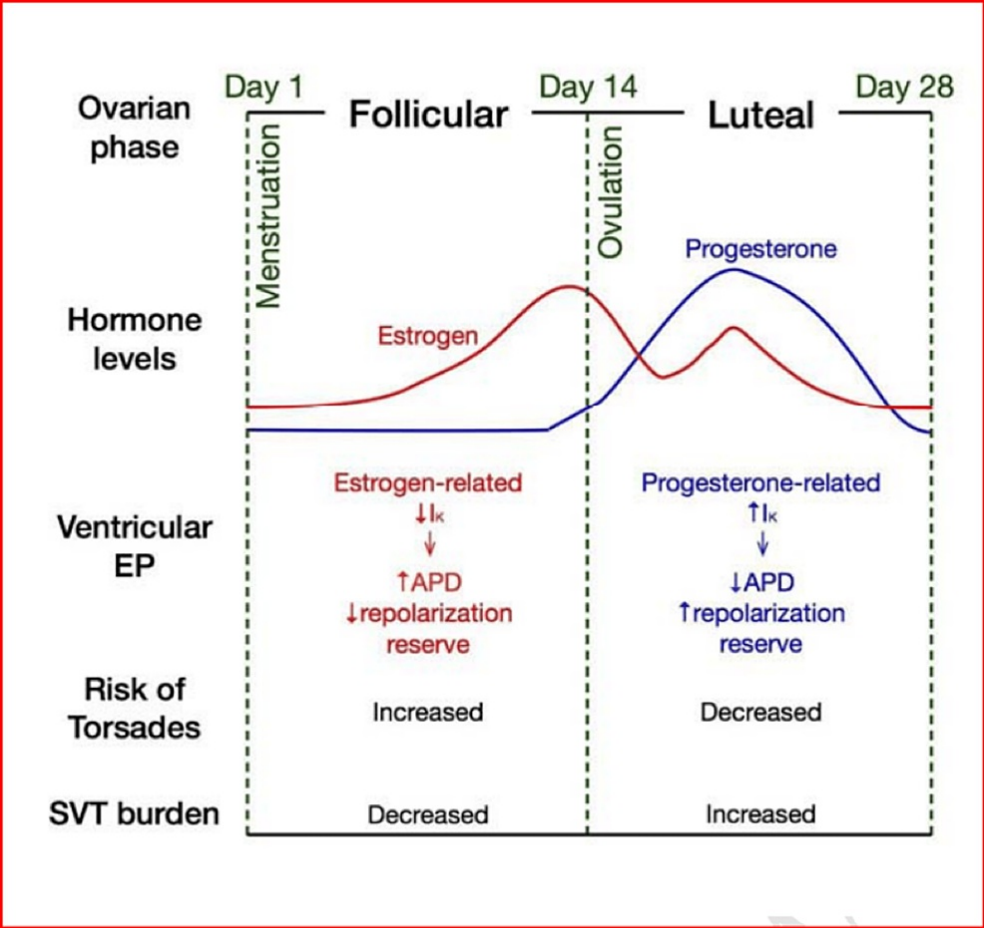
Female ovarian cycle with associated electrophysiological changes and susceptibility to arrhythmia. Follicular-phase properties are in red; the luteal-phase properties in blue. Reproduced under the terms of the Creative Commons attributions license [72].

Human data show that female ventricles express a variety of K+ channel subunits less strongly that contribute to repolarization than male ventricles. Women show cyclic variations in QTc, with QTc longer in the follicular than the luteal phase, after autonomic blockade. These are due to the modulating effects of estrogen and progesterone on the cardiac potassium channels during the ovarian cycle, which alter ion currents through nongenomic effects: testosterone and progesterone increase, while estrogen decreases, the repolarizing currents. Thus, increased estrogen/progesterone ratios during the follicular phase decrease repolarizing currents, prolonging APD and QTc [4]. Estrogen causes an increase in the QT interval, whereas progesterone decreases the QT interval [2]. Figure 5 shows the female ovarian cycle with related electrophysiological changes and arrhythmia susceptibility.

**Figure 6:**
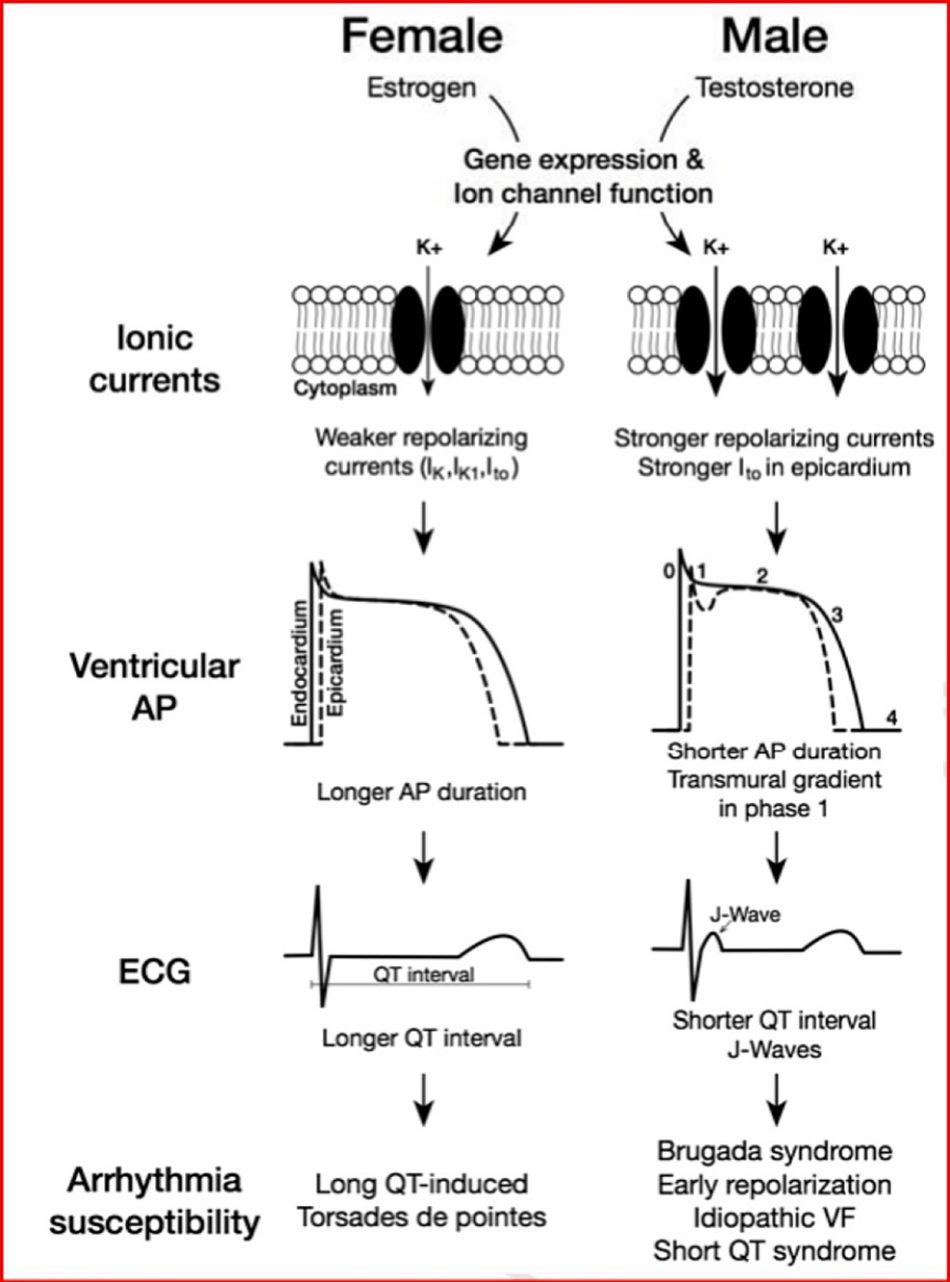
Sex differences in the ventricular cardiomyocyte electrophysiology. I_K_: delayed rectifier K+ current; I_to_: transient-outward K+ current Reproduced under the terms of the Creative Commons attributions license [72].

Figure 6 shows the sex differences in the ventricular cardiomyocyte electrophysiology.

Mortality risk in LQTS can vary by age, sex, QTc interval, genotype, and history of symptoms. A narrowly defined subgroup of LQTS patients, those over age 20 with no history of syncope prior to age 20 and a QTc of less than 500 ms, have an excess death rate of 0.5-2.0 per thousand per year [73]. This shows that there is an association between prolonged QT interval and increased risk of cardiovascular and sudden cardiac death. The QT-interval length is a determinant of mortality in the general population [74]. Women who have LQTS are more likely to have a higher mortality rate from the condition, especially during menstruation and shortly after giving birth [75].

Challenges and limitations in diagnosis of LQTS

**Figure 7:**
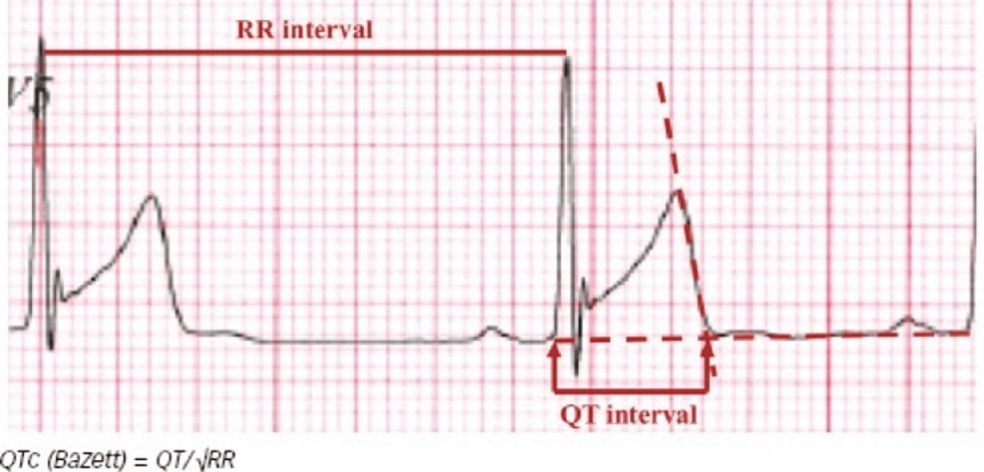
Measurement of the QT interval. Reproduced under the terms of the Creative Commons attribution license [76].

LQTS can be difficult to diagnose because defining the QT interval itself is a tough task. Studies have suggested that the ability of cardiologists and even heart rhythm specialists to accurately measure the QTc is suboptimal [76]. The first difficulty is to define the end of the T wave. This is usually done by drawing the tangent line to the steepest part of the descending portion of the T wave, chosen in a lead where the T wave has the greatest amplitude, taking its intercept with the isoelectric line as the end of the T wave (Figure 7).

Another problem is that there may be no little or no prolongation of QT interval in acquired LQTS (aLQTS), unless the patient is exposed to triggers that prolong the QT interval, such as medications, hypokalemia, and bradycardia. Some cases of aLQTS have underlying cLQTS, which could be unmasked due to the exposure to triggers. Sometimes, QTc remains prolonged despite the removal of triggers, suggesting the presence of an underlying genetic substrate [77]. One study demonstrated that the QTc measured in the absence of triggering factors of aLQTS cases is shorter than that of cLQTS patients, but it is significantly longer than that of individuals without cLQTS [77]. Therefore, even a moderate increase in the QT interval in the absence of triggers should raise suspicions for cLQTS. However, even in patients with a normal QT interval in the absence of the QT-prolonging triggers, 23% had cLQTS-causing mutations [77]. This poses a problem as to what should be the cut-off value for a normal QT interval to guide physicians to diagnose LQTS even before an actual QT prolongation on exposure to the triggers that could lead to life-threatening TdP and ventricular fibrillation.

Moreover, diagnosis is more difficult in athletes because the QT interval is prolonged by training, and the extreme bradycardia frequently observed in athletes makes the QT correction formula less accurate [76]. There are multiple formulas for calculating the cQT interval when the heart rate changes. The most widely used formula is Bazett’s formula (QTc = QT /√RR) using the RR interval preceding the QT interval measured [78]. At slow heart rates, which frequently occur in athletes (due to a change in the automatic balance with a lower sympathetic activity and a higher vagal tone at rest), the QTc interval may be underestimated if Bazett’s formula is used [76]. Thus, the heart rate should be increased to normal for calculating the QT interval in athletes. There is a limitation in genetic testing for aLQTS as multiple genes affect the QT interval duration. Genes that are commonly implicated in cLQTS, such as KCNQ1 and KCNH2 genes, can be detected, but genes responsible for the rarer variants of cLQTS are difficult to analyze. Although their probability of causing cLQTS is very low (<1%), we cannot exclude the possibility that some genotype-negative aLQTS patients may have mutations in these genes or in others not yet identified [77].

The diagnosis of cLQTS warrants genetic testing of family members to identify those who are at risk of complications of LQTS. Having identified the predisposing mutation in the patients, "cascade screening" in their families will rapidly and inexpensively identify additional mutation carriers and prevent avoidable risks of life-threatening arrhythmias [77]. cLQTS is an autosomal dominant disorder, so every individual with this disorder has a 50% chance of transmitting it to their offspring with variable penetrance. Prenatal testing for a pregnancy at increased risk and preimplantation genetic testing is possible once the pathogenic variant(s) have been identified in the family [79]. Thus, the family should be counseled regarding genetic testing during pregnancy so that the diagnosis can be made beforehand and can be managed likewise. Parents of the affected individuals can suffer from distress due to the unpredictable and lethal nature of the disease. Parents of the carrier children, who are at risk for symptoms of LQTS, remain preoccupied with the disease for a long time (at least for 18 months) [80]. Therefore, this psychological distress of the parents should be addressed appropriately. One of the ways is to give these parents adequate information regularly and keep them informed on the most recent developments in the management of LQTS [80]. This will help them understand the nature of the disease and can lead to diminished levels of distress they feel of having a child with a disease that can lead to life-threatening arrhythmias.

## Conclusions

This study offers a thorough examination of LQTS, illuminating its biology, clinical signs, and treatment options. The study emphasizes the complex interaction among ion channel malfunction, genetic susceptibility, and triggering conditions resulting in potentially fatal arrhythmias. This research advances our knowledge of LQTS by illuminating the underlying mechanisms and lays the road for more precise diagnostic procedures and individualized therapeutic strategies. The results highlight the value of early detection, risk stratification, and tailored interventions to reduce the hazards that are involved. Although improvements in genetic testing and pharmacology present promising directions, further study is required to elucidate the full range of LQTS variants and improve treatment regimens. In the end, this study offers significant knowledge that can direct healthcare professionals in optimizing patient treatment and lowering the morbidity and mortality related to LQTS.
